# Innate Immune Mechanisms to Protect Against Infection at the Human Decidual-Placental Interface

**DOI:** 10.3389/fimmu.2020.02070

**Published:** 2020-09-10

**Authors:** Regina Hoo, Annettee Nakimuli, Roser Vento-Tormo

**Affiliations:** ^1^Wellcome Sanger Institute, Cambridge, United Kingdom; ^2^Centre for Trophoblast Research, University of Cambridge, Cambridge, United Kingdom; ^3^Department of Obstetrics and Gynecology, School of Medicine, Makerere University, Kampala, Uganda

**Keywords:** innate immunity, uterine-placental interface, trophoblast, decidua, vertical transmission

## Abstract

During pregnancy, the placenta forms the anatomical barrier between the mother and developing fetus. Infectious agents can potentially breach the placental barrier resulting in pathogenic transmission from mother to fetus. Innate immune responses, orchestrated by maternal and fetal cells at the decidual-placental interface, are the first line of defense to avoid vertical transmission. Here, we outline the anatomy of the human placenta and uterine lining, the decidua, and discuss the potential capacity of pathogen pattern recognition and other host defense strategies present in the innate immune cells at the placental-decidual interface. We consider major congenital infections that access the placenta from hematogenous or decidual route. Finally, we highlight the challenges in studying human placental responses to pathogens and vertical transmission using current experimental models and identify gaps in knowledge that need to be addressed. We further propose novel experimental strategies to address such limitations.

## Introduction

The human placenta is the temporary extra-embryonic organ that is present only during pregnancy and is the anatomical boundary between the mother and fetus. It has a range of functions including transport of nutrients and gases, and hormonal production (1). The placenta forms a physical, selective barrier between the maternal and fetal circulations, preventing transfer of pathogens. The uterine mucosal lining, the endometrium, is transformed into the decidua during early pregnancy (2). A range of innate immune mechanisms can respond to pathogens in both the decidua and the placenta (3, 4). The maternal-fetal interface is a protective barrier against pathogens, but some pathogens can transfer from the mother to fetus by different routes and cause fetal infection (3, 4).

**Figure 1:**
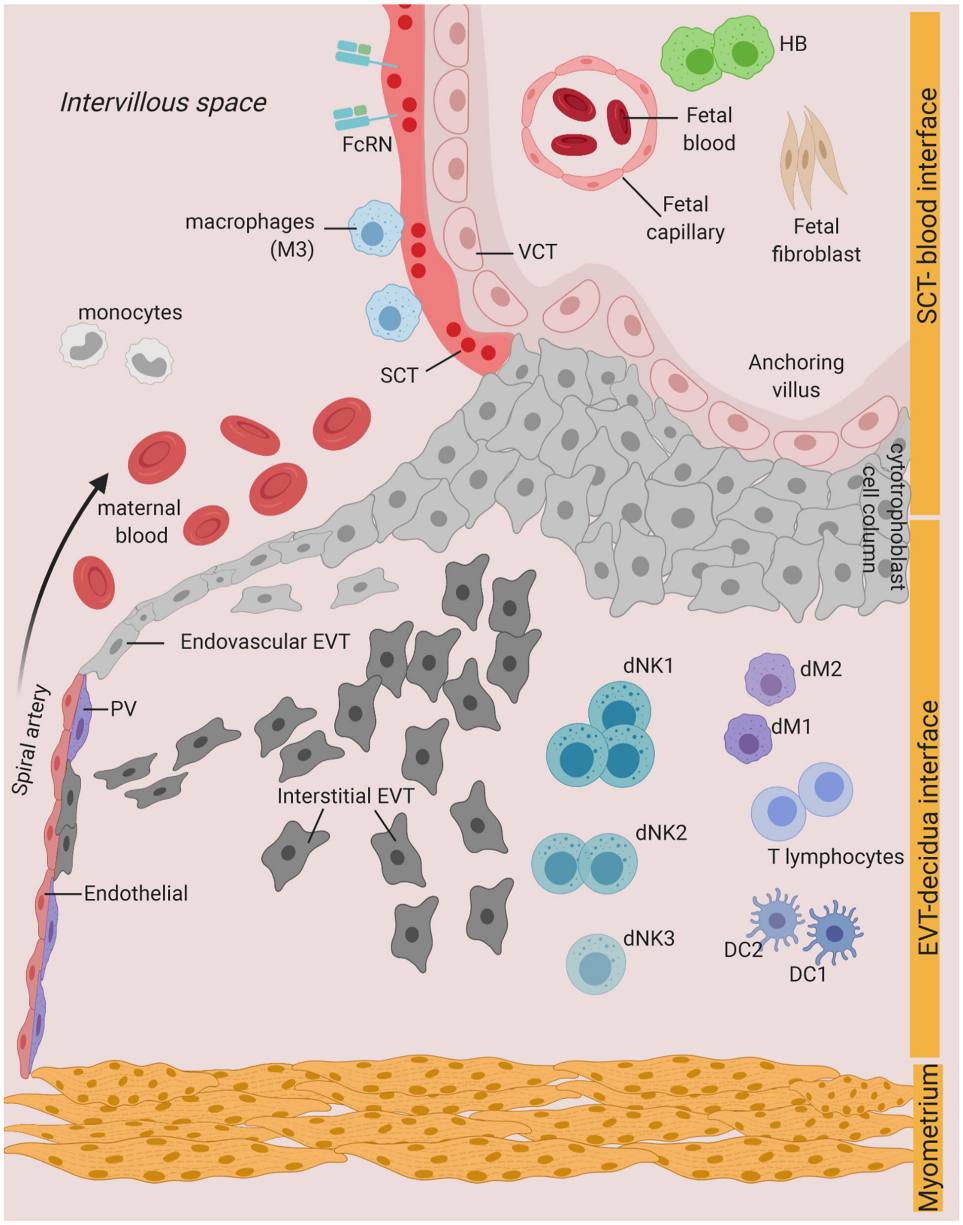
Possible infection and vertical transmission route at the maternal-fetal interface. Illustration representing the anchoring placenta villi of early pregnancy, with onset of maternal blood circulation bathing the intervillous space. SCT-blood interface represents the SCT barrier exposed to maternal blood and immune cells. EVT-decidua interface represents the interface between EVT and maternal decidua cells. Major cell types of placenta trophoblast and decidua from Vento-Tormo et al. (10) are represented. SCT, syncytiotrophoblast; VCT, villous cytotrophoblast; EVT, extravillous trophoblast; DC, dendritic cell; dNK, decidua Natural killer cells; dM, decidua macrophages; HB, Hofbauer cell; PV, perivascular cells; FcRN, neonatal Fc receptor. Figure is created by BioRender.com.

Vertical transmission during pregnancy can occur on distinct boundaries between the mother and the fetus: (i) the intervillous space (IVS), where placental villi is in direct contact with the maternal blood, (ii) the implantation site or decidua basalis, where maternal cells are in direct contact with the invading fetal trophoblast, and (iii) the fetal membranes, which are in direct contact with the uterine cavity (5). Defense mechanisms in the cervix, such as the production of mucus and antimicrobial peptides (AMP), limit ascending infection from pathogens present in the lower genital tract, that otherwise may access the uterine cavity (6). However, some pathogens can escape antimicrobial strategies at the cervix and ascend to the uterus, where they can bypass the fetal membranes and lead to the inflammation of the membranes- also known as chorioamnionitis- and infection of the amniotic fluid (7, 8). Pathologic and immune features of chorioamnionitis and intra-amniotic infection are generally associated with bacterial invasion and inflammation [refer to (8, 9) for a comprehensive review on these mechanisms]. Here, we focus on infections and innate immune mechanisms at the uterine-placental interface—cases (i) and (ii) (Figure 1).

Infections at the uterine-placental interface are commonly associated with viruses, parasites and few bacteria (Table 1). Viral pathogens such as human cytomegalovirus (HCMV), Zika (ZIKV), and rubella virus are the most common vertically transmitted pathogens through the decidual-placental interface (Table 1) (26, 27). Non-viral pathogens, such as *Toxoplasma gondii* and *Listeria monocytogenes*, can cross the placental barrier via cell-to-cell transmission (Table 1) (28, 29). Fetal infection can result in various forms of congenital anomalies in humans (Table 1). Understanding the pathogenic mechanisms used by infectious agents is central to preventing vertical transmission and controlling infection during pregnancy.

**Table 1:** Vertically transmitted pathogens with clinical evidence from natural human infection.

**Species**	**Lifestyle**	**Life cycle and pathogenesis**	**Clinical manifestations**	**Evidence of cellular tropism in the human placenta or decidua by histology or PCR**	**References**
*Chlamydia trachomatis*	Intracellular bacteria	Formation of reticulate body inside host cell allows for rapid replication Conversion of reticulate body to elementary body inside host cell promotes the release of infectious bacteria to neighboring cell	Ectopic pregnancy, stillbirth, preterm labor, blinding corneal injury in neonates, neonatal pneumonia	Whole placenta, glandular epithelial cells, unidentified decidual cells	(11)
Group B Streptococcus (*Streptococcus agalactiae*)	Non-motile, extracellular bacteria	Beta-hemolytic Strong adherence to epithelial layer Able to form biofilm	Neonatal GBS (sepsis and meningitis), preterm birth	Amniotic epithelium, amniotic fluid, chorion, decidua	(12)
*Listeria monocytogenes* (Listeriosis)	Motile intracellular bacteria	Utilize two bacterial surface proteins (internalin A and B) to invade various non-phagocytic cell types Able to escape phagosome-mediated lysis and multiply rapidly in host cytoplasm Able to spread to adjacent cell through host cell actin polymerization	Spontaneous abortion, stillbirth, preterm labor	Placenta trophoblast	(13)
*Coxiella burnetii* (Q fever)	Intracellular bacteria	Able to escape phagosome-mediated lysis in macrophage	Spontaneous abortion, preterm delivery, fetal death	Placenta (unknown cell type)	(14)
*Treponema pallidum*	Motile spirochaete, extracellular bacteria	Able to transverse tight-junction between endothelial cells Highly motile	Congenital syphilis	Placenta (unknown cell type)	(15, 16)
*Toxoplasma gondii* (Toxoplasmosis)	Intracellular parasite	Able to infect and replicate within various host cell types Able to switch between non-motile (for replication) and motile state (for egress and invasion into new host cell)	Congenital toxoplasmosis, stillbirth	Placenta trophoblast	(17)
*Trypanosoma cruzi* (Chagas)	Intracellular and extracellular parasite	Able to propagate in various host cells and escape Progeny released by host cells are motile, and able to infect distal tissue or organs	Stillbirth, preterm labor	SCT, villous stroma, placenta basal plate	(18)
Herpes simplex virus 1, 2 (HSV-1/2)	dsDNA virus	Able to cross through skin lesions and epithelial mucosal cells Poor antibody neutralization to viral glycoprotein D (gD) Vertical transmission rate is very low	Spontaneous abortion, intrauterine growth restriction, preterm labor, neonatal herpes	Decidua	(11)
Human cytomegalovirus(HCMV)	dsDNA virus	Easily transmitted through bodily fluid Poor antibody neutralization to viral glycoprotein B (gB) Can establish lifelong latency in myeloid cells	Variable; neonatal neurodevelopmental damage and hearing loss	VCT, decidua, amniotic membrane	(19)
Rubella	ssRNA virus	Able to enter the lymphatic system from the respiratory tract Can lead to a systemic infection Viral capsid can evade host immune recognition	Significant birth defects, neonatal deafness, miscarriage	Placenta basal plate and endothelial cells	(20)
Parvovirus B19	ssDNA virus	Spread through respiratory droplets Preferential tropism for human erythroid progenitor	Fetus is usually unaffected, may result in severe fetal anemia	Whole placenta, placenta villi	(21)
Varicella zoster virus (Chicken pox)	ssDNA virus	Vertical transmission is very rare and only happens in primary infection	Congenital varicella syndrome, intrauterine growth restriction, low birth weight	No evidence, but chronic villitis has been described	(22)
ZIKA virus (ZIKV)	ssRNA virus	Mosquito borne infection transmitted from blood meal Preferentially to invade blood monocytes	Congenital fetal anomalies (microcephaly), miscarriage, stillbirth	Whole placenta, amniotic epithelium, VCT, Hofbauer cells, decidual macrophages, decidual fibroblast	(23–25)

How the innate immune cells and mechanisms in the placenta and the uterus recognize and respond to protect both the fetus and mother remains controversial due to technical and ethical constraints. However, there are several different models currently used to interrogate the uterine-placental interface in pregnancy. Firstly, mice are frequently used as a pregnancy model for infection. Although the murine models have provided important insights into the pathogenesis of various infection agents in the context of pregnancy, there are still limitations with this approach. The anatomy of placentation, length of gestation, and use of inbred strains, make extrapolation to humans problematic (30, 31). Secondly, a range of human trophoblast and choriocarcinoma cell lines are used as *in vitro* models for infection with pathogens. In contrast to the first trimester trophoblast *in vivo*, these cell lines do not recapitulate normal human trophoblast characteristics such as expression of the human leukocyte antigen (HLA) class I and methylation of *ELF5* (32, 33). Thirdly, human primary placental explants are frequently used. The syncytium dies rapidly in these cultures and it is virtually impossible to standardize the types of villi sampled (30). Therefore, these *in vitro* experimental factors should be taken into careful consideration when interpreting studies of infection of trophoblast.

In this review, we cover the innate immune features of the decidual-placental interface throughout gestation. We identify the gaps in knowledge and highlight the limitations of current studies and experimental models. Finally, we discuss novel experimental strategies for understanding how infection affects pregnancy in humans.

### Physiology of the Placenta Throughout Gestation

The trophoblasts of the placenta are the barrier between fetal and maternal tissues. They are derived from the trophectoderm, the outer layer of the blastocyst that forms an inner mononuclear layer with an outer primary syncytium following implantation (34). The trophoblast in contact with the maternal cells can be: (i) syncytiotrophoblast (SCT), a single layer multinucleated, syncytial layer formed by fusion of the underlying villous cytotrophoblast (VCT), and (ii) extravillous trophoblast (EVT), that invade from the cytotrophoblast shell and anchoring villi into the transformed maternal endometrium, the decidua (2).

The function of EVT is to transform the uterine spiral arteries so that maternal blood is delivered to the intervillous space at low pressure. The arteries are surrounded by interstitial EVT that destroys the smooth muscle cells of the arterial media, known as “fibrinoid” change (35, 36). Subsequently, endovascular EVT (eEVT) moves down the spiral arteries from the placenta-decidua boundary (35). These eEVT form a plug of cells, limiting surges of arterial blood from damaging the delicate villi. EVT invasion transforms the arteries to support optimal regulation of blood flow into the placenta during fetal development (36). The plugs dissipate between 8 and 10 weeks of gestation when the full hemochorial circulation is established (37). Maternal blood then flows into the IVS, and establishes direct contact with the SCT allowing for proper nutrient and gas exchange between the mother and the fetus.

## Hofbauer Cells: the Tissue Resident Immune Cells of the Placenta

Hofbauer (HB) cells are fetal macrophages of the human placenta (38). HB cells can be detected in the placental villous stroma as early as 3 weeks post-conception and are present throughout pregnancy (1, 39). They are likely to have a variety of functions including control of villous remodeling and differentiation, hormonal secretion, and trophoblast turnover (1, 40). Several lines of evidence have led to the postulation that HB cells may have a role in infection during pregnancy. HB cells with ZIKV viral particles detected intracellularly have been shown (41, 42). Human immunodeficiency virus 1 (HIV-1) has also been detected in HB cells from first trimester infected placenta (43). Whether the HB cells can serve as a reservoir or limit virus replication is still unknown. Isolated HB cells from healthy term placenta show elevation of pro-inflammatory cytokines such as IL-6, MCP-1, IP-10, and IFN-α upon *in vitro* infection with ZIKV (44). HB cells from the first trimester placenta are also permissive for ZIKV infection and replication (23). However, this must be interpreted with caution because *in vitro* culture of HB cells do not entirely recapitulate the complexity of villous stromal microenvironment, such as presence of hormone and growth factors, all of which will influence the function and activity of HB cells (45).

## Maternal Blood and SCT Interface

The SCT is the barrier between maternal blood and the placental core as it separates the IVS from the underlying fetal villous stroma. Blood-borne pathogens such as viruses and parasites can potentially be transmitted through the SCT barrier (Figure 1).

**Figure 2:**
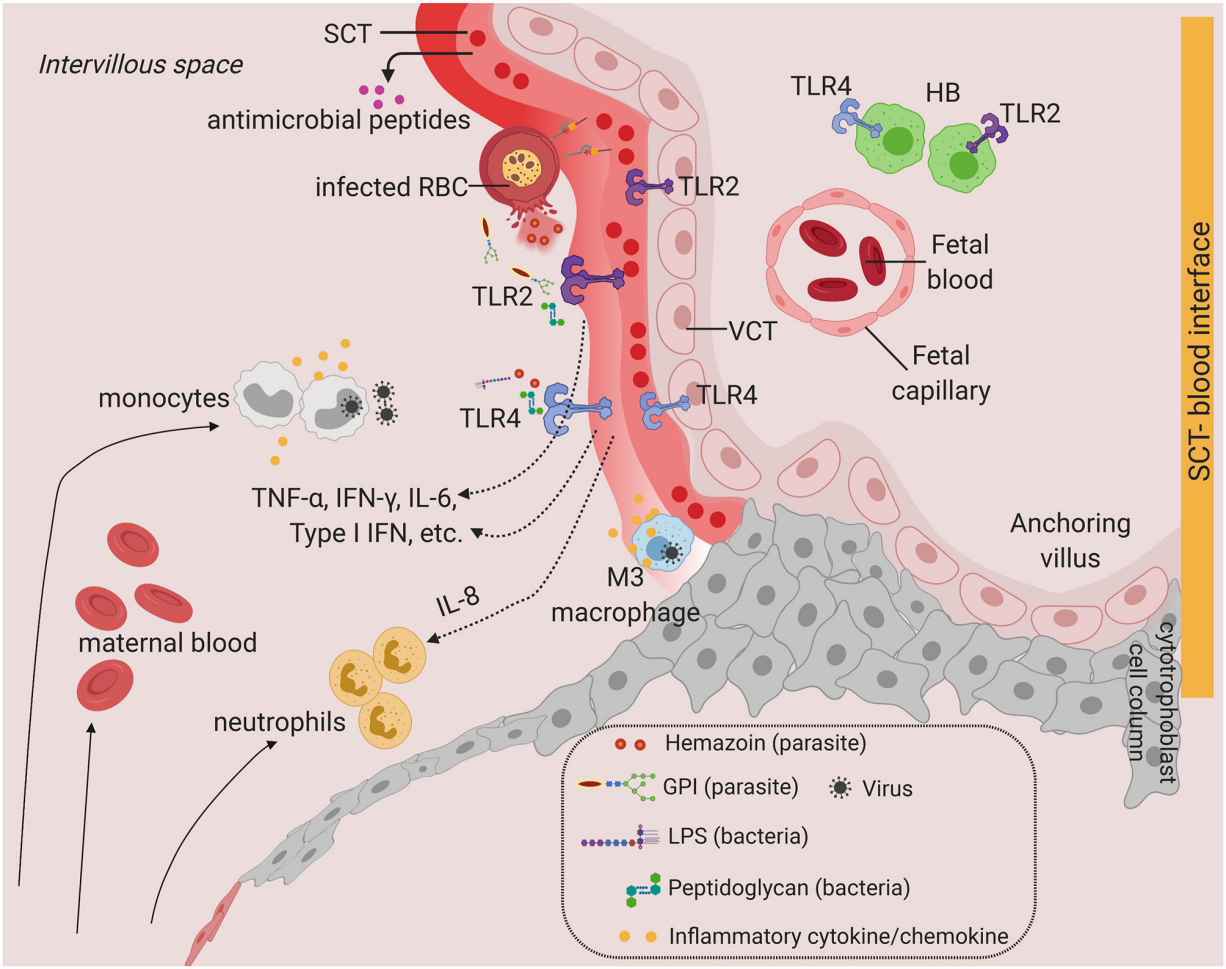
Toll-like receptors and potential inflammatory response at the SCT-blood interface. Predominant TLRs found in the human placenta from early and term pregnancies. TLR2 and TLR4 are expressed in human placenta SCT, VCT, and in HB cells. Infiltration of infected maternal blood, infected immune cells, or release of pathogenic determinant such lipopolysaccharide (LPS), peptidoglycan, or parasite materials such as hemozoin or GPI (glycosylphosphatidylinositol) into the IVS will activate TLR-mediated signaling, leading to the production of a wide range of cytokines and chemokines. Severe infection is characterized by massive immune cell infiltration including monocytes and neutrophils from systemic circulation and overproduction of inflammatory cytokines upon TLR activation. This may lead to SCT inflammation and damage. SCT also secretes antimicrobial peptides as innate immune mechanisms. Figure is created by BioRender.com.

How can pathogens cross the SCT barrier and the VCT to infect the villous stroma? Although the SCT is an efficient barrier due to its stiff, highly dense actin cytoskeleton network and continuous membrane (46), the syncytium undergoes continuous breaks or gaps and dynamic repair processes (47). Breaks in the syncytium could potentially lead to transmission of pathogens into the underlying VCT. Our recent work showed that a novel population of maternal macrophages (M3) is associated with the SCT in early pregnancy and might be involved in repairing the breaks in the syncytium (10). It is intriguing that M3 macrophages infected with intracellular pathogens could possibly gain access to the underlying VCT via the syncytial breaks (Figure 2).

Only a few viral entry receptors on the SCT are described. Notably, the SCT lacks expression of ZIKV entry receptors, Axl, and Tyro3 (48) and the HCMV entry co-receptor integrin α/β (49). This is further supported by the transcriptomic expression of viral receptors in placental cells (10, 50, 51). Expression of surface receptors commonly used by ZIKV such as *AXL* and *HCMV* such as *NRP2* and *PDGFRA* are lowly expressed by the SCT (50). In addition, there is minimal co-expression of ACE2, the receptor gene for human severe acute respiratory syndrome coronavirus 2 (SARS-CoV-2), and TMPRSS2, the viral spike protein serine protease gene (50, 52). In line with this, there is no conclusive and direct evidence of vertical transmission of SARS-CoV-2 in a placenta from a healthy individual. There are some reports showing SARS-CoV-2 is predominantly localized at the SCT of the second trimester placenta (53, 54) and can lead to severe inflammatory infiltrate in the IVS (55). However, these findings are presented in a very small number of patients with severe disease or pre-existing pregnancy complications (54, 55).

Alternative transplacental mechanisms have been postulated at the syncytial barrier. Neonatal Fc receptor (FcRn) is expressed on the apical surface of the SCT and functions to selectively transport maternal IgG (56). FcRn could be exploited by certain viruses to enter the placenta including ZIKV, HIV-1, and HCMV (19, 57, 58). Transferrin receptor 1 (TfR1) is expressed on the apical end of the SCT, and functions as the primary iron transporter into the basal side of the SCT to provide sufficient iron stores into fetal circulation (59). TfR1 has been associated with viral entry into a broad host cell range, including Hepatitis C virus (60, 61) suggesting a possible mechanism of viral transport across the SCT barrier. Some pathogens, although unable to cross the SCT barrier, can still adhere to the syncytium and cause further pathology. For instance, *Plasmodium falciparum* infected red blood cells can bind with high affinity to chondroitin sulfate A expressed on the SCT, resulting in local inflammation, syncytial breaks, and damage (62–64).

Although the SCT is an effective barrier to most pathogens, local inflammation, tissue damage, and FcRN or TfR1-mediated viral entry at the SCT can potentially allow pathogen to breach the syncytial barrier, giving opportunity for transmission from maternal blood into placental villi (Figure 2).

## Maternal Decidua and EVT Interface

During the first trimester of pregnancy, fetal EVT invades deeply into the uterus. The decidua basalis, the region located at the implantation site, is populated at this time by a distinctive subset of innate lymphocytes, decidual Natural Killer cells (dNK), which constitute up to 70% of leukocytes. We have identified three major populations of dNK by single-cell RNA-sequencing with unique phenotypes and functions in early pregnancy (10). In addition, there are populations of decidual macrophages (dMs) (~20%), conventional dendritic cells (DCs) and small proportions of T cells (~10–15%), whereas B cells, plasma cells, mast cells, and granulocytes are virtually absent (10) (Figure 1). The proportion of immune cells will vary throughout pregnancy, with an increase in the proportion of T cells at term (51).

Systemic infections will reach all organs including the decidua. Whether pathogens can also access the decidua via the cervix is still unclear. *Chlamydia trachomatis*, a common sexually-transmitted intracellular bacteria, was detected in glandular epithelial cells and unidentified decidual cells in decidual biopsies (11). This suggests the possibility of infections ascending and spreading from cell-to-cell from the lower genital tract into endometrial glands and vascular endothelium. The decidua basalis is in close contact with fetal cells and the maternal vasculature (Figure 1). First trimester dMs and decidual stromal cells are susceptible to ZIKV infection and replication *ex vivo* (23). Hence, infection could possibly spread from infected maternal immune and non-immune cells at the decidua, into uninfected VCT in the columns of the anchoring villi, and finally into the fetal compartment. However, this is likely to be limited to certain microorganisms which are capable of cell-to-cell spread, have an intracellular host niche, and are able to escape host innate defense mechanisms (Table 1).

HCMV, the most common cause of congenital infection, is mostly reported to infect from the decidua (11, 65). Women with primary HCMV infection and first pregnancy are more likely to transmit the virus to their fetus, compared to multiparous women with previous infection and demonstrable antibodies (66–68). Low affinity maternal antibodies against HCMV correlate with higher viral loads detected in the decidua, whereas patients with intermediate to high neutralizing antibodies have minimal viral replication (65), suggesting that maternal immunity against HCMV reduces risk of vertical transmission. HCMV protein was also detected in a range of cells within the decidua including endothelial, decidual stromal cells, DCs and macrophages (11, 65), suggesting that that infected maternal leukocytes could initiate transmission through contact and infection of endothelial cells that line decidual blood vessels.

Despite the evidence of decidual infection, the mechanism of vertical transmission for HCMV is still in debate. dNKs have been proposed to play a protective role against HCMV infection through several mechanisms including modulation of their cytotoxic effector function (69) and the interactions between the killer-cell immunoglobulin receptors (KIRs) expressed by dNK and HLA molecules expressed in the infected cells (70, 71). Activating KIR2DS1 by dNKs has been demonstrated to be more cytolytic against HLA-C2 HCMV-infected maternal decidual stromal cells (70). Similar cytotoxic response was also observed when peripheral blood NK cells expressing KIR2DS1 were exposed to HCMV-infected fibroblasts (71). Hence, this implies that in the decidua, dNKs are capable of eliminating harmful infection depending on the combination of KIR/HLA interactions between dNK and infected cells. dNKs are also able to control HIV-1 infection *in vitro* through production of IFN-γ (72). The role of dNK in controlling viral infection may protect against potential risk of vertical transmission from the decidua.

## Transmembrane Pattern Recognition Receptors: Toll-Like Receptors

Pattern recognition receptors (PRR) are encoded in the germ-line and recognize specific, conserved pathogen-associated molecular patterns (PAMPs). These include Gram-negative bacteria lipopolysaccharide (LPS), Gram-positive bacteria lipoteichoic acids, lipoprotein, DNA, RNA, glucans, and peptidoglycans (73, 74). Pathogen recognition is not only an essential component of the innate immune response against infection, but also plays an important role in bridging the innate and adaptive systems by Toll-like receptors (TLR) activation of antigen presenting cells by up-regulation of major histocompatibility complex (MHC) and co-stimulatory molecules (75).

TLRs, the most studied family of PRR, are type I transmembrane proteins with large extracellular domains containing leucine-rich repeats that are expressed at the cell surface or intracellularly (76). Each TLR recognizes distinct PAMPs, leading to the activation of the transcription factor NF-κB and/or the interferon-regulatory factor (IRF) family, and the production of a wide range of cytokines and chemokines, including type I IFNs (76, 77). TLRs are expressed by immune cells (macrophages, DCs, and B cells) as well as non-immune cells (fibroblasts and epithelial cells) (74).

### TLRs at the Human Uterine-Placental Interface

**Figure 3:**
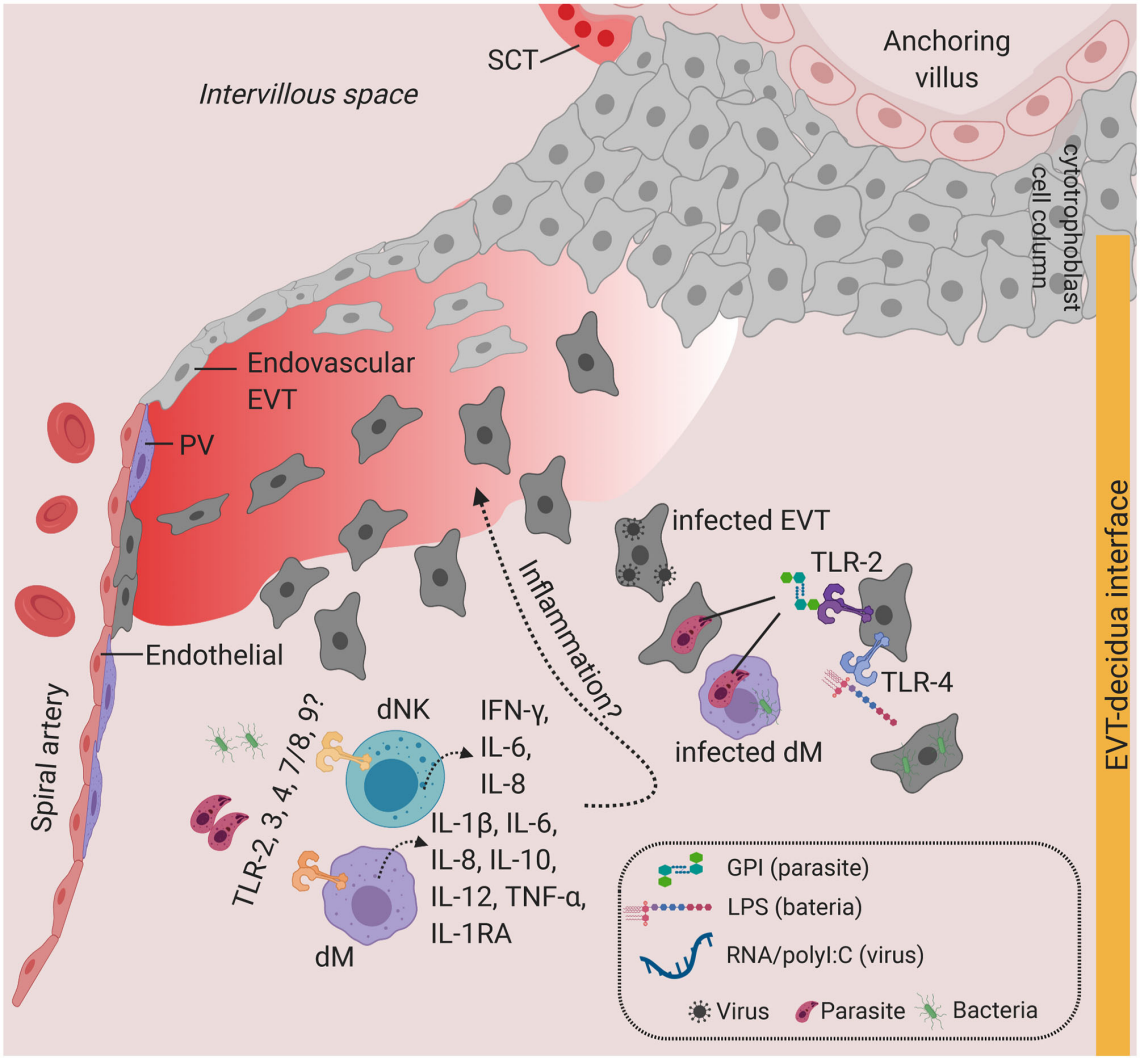
Toll-like receptors and potential inflammatory response at the decidua. Predominant TLRs found in the human placenta from early and term pregnancies. TLR2 and TLR4 are expressed in EVT. dM and dNK also express a wide range of TLR families, where stimulation of TLR agonists lead to the production of a variety of cytokines and chemokines. Infiltration of infected cells and release of PAMPs in the decidua, which will activate TLR-mediated signaling. Overproduction of inflammatory cytokines at the decidua may lead to local inflammation. Figure is created by BioRender.com.

Expression of TLRs is dynamic and changes in response to different pathogens and cytokines (74). TLR2 (which recognizes bacterial proteoglycan) and TLR4 (which recognizes bacterial LPS) are the most well-studied, with immunohistochemical evidence of expression in healthy primary SCT at term (78–80). In contrast, in the first trimester, TLR2 and TLR4 proteins are expressed in VCT and EVT, but minimally in SCT (81, 82) (Figure 3). There is therefore variation in TLR2 and TLR4 expression in the different trophoblast lineages across pregnancy. Why and how such dynamic regulation of TLR expression occurs during gestation requires further investigation in a broader range of human placental samples (different donors, gestation stages, genetic background, sampling regions). It is likely that alteration in cytokines profiles in the microenvironment as pregnancy progresses (83) may result in the variation in the expression of TLRs in the placenta. Current evidence is only limited to *in vitro* TLR2/4 stimulation studies using placental explants and primary first trimester trophoblast cells, which drives the expression of pro-inflammatory cytokines IL-6, IL-8, TNF-α, and IFN-γ (78, 80, 81).

**Figure 4:**
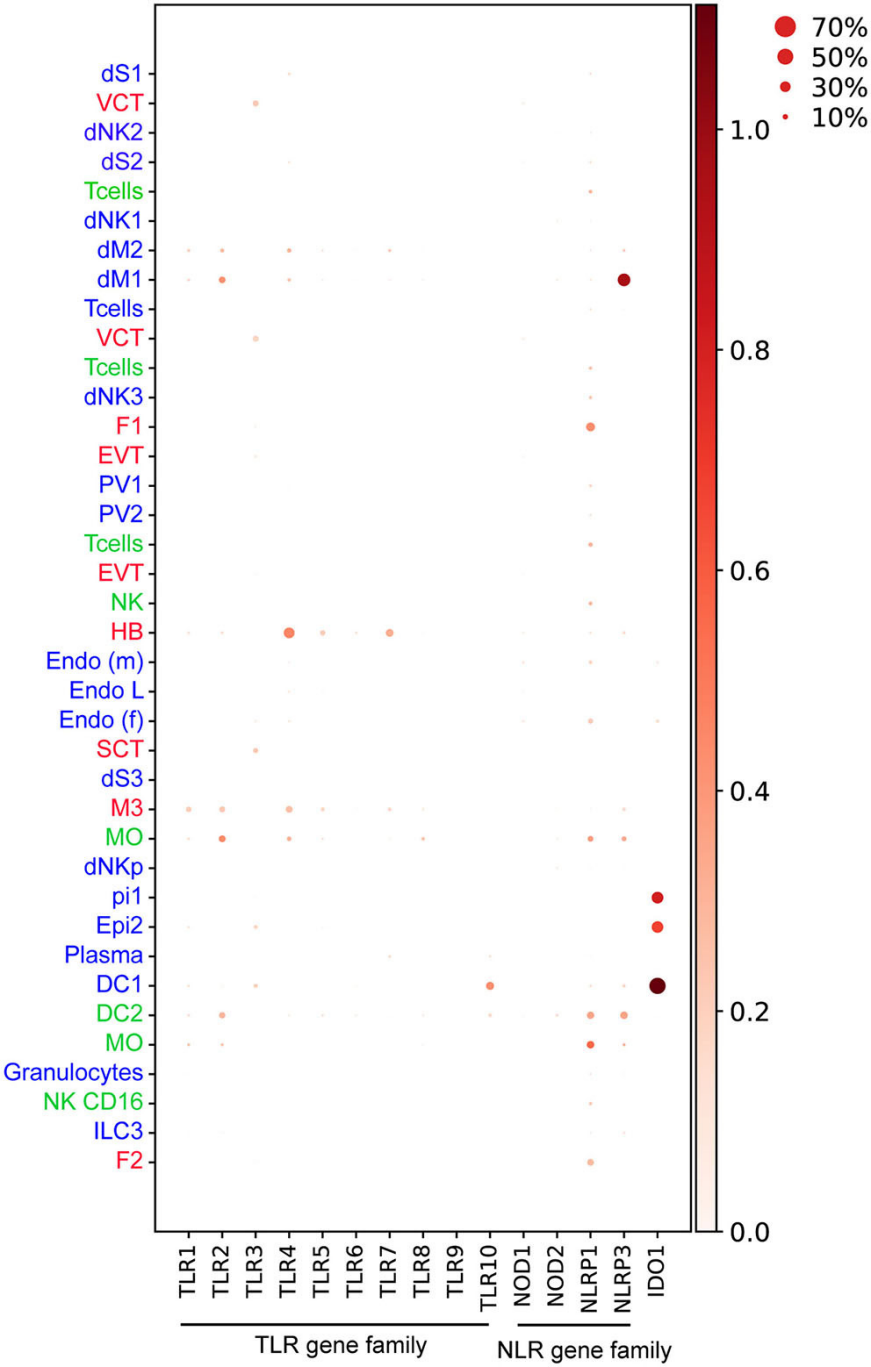
Dotplot representing normalized and log transformed values expression of TLR (*TLR1-10*), NLR genes (*NOD1, NOD2, NLRP1, NLRP3*) and *IDO1* at steady state in early pregnancy from Vento-Tormo et al. (10). Origin of cell types from placenta (red), decidua (blue), and maternal blood (green) are labeled as differences in font color. Dot size represents the fraction of cells from a certain cluster expressing a gene and color scale represents normalized log transformed expression of the gene in that cluster. dS, decidua stroma; F, fibroblast; MO, monocyte; Endo, endothelial; Epi, epithelial; SCT, syncytiotrophoblast; VCT, villous cytotrophoblast; EVT, extravillous trophoblast; DC, dendritic cell; dNK, decidua Natural killer cells; dM, decidua macrophages; HB, Hofbauer cell; PV, perivascular cells. Figure is created by BioRender.com.

TLR2 and TLR4 proteins are expressed in HB cells, confirmed by co-expression of CD68 in healthy term placentas (78). In early pregnancy, our findings indicate that only *TLR4* but not *TLR2* transcripts are expressed in steady-state HB cells (10) (Figure 4). Enhancement of IL-6 and IL-8 secretion upon stimulation of isolated first trimester HB cells with TLR4 agonist, LPS (84), does suggest a role for TLRs on HB cells in bacterial recognition and placental inflammation during early pregnancy. HB cells are postulated to have a role in viral replication (41, 42), however evidence on the expression and function of viral nucleic acid sensing receptors TLR3, TLR7, TLR8, and TLR9 in HB cells is lacking. Our findings show that *TLR7*, which recognizes viral single-strand RNA (ssRNA) (85) is expressed in steady-state HB cells (Figure 4) (10).

Other TLRs have also been shown to be expressed in decidua cells. dMs and dNKs isolated from first trimester pregnancies show steady state level expression of *TLR1-9* transcripts and respond to a broad range of PAMPs, including heat-killed bacteria, microbial membranes, and nucleic acids (86). Stimulating primary dMs with these PAMPs produces high levels of TNF-α, IL-1β, IL-6, IL-8, IL-12, IL-10, and IL-1RA, whereas dNKs secrete IL-6, IL-8, and IFN-γ (86). This study suggests that, in addition to the physiological roles of dMs and dNKs in accommodating the uterus for placentation, dMs and dNKs may play a role in pathogen recognition and antimicrobial response via activation of TLR signaling (Figure 3). The extent to which subsets of dMs or dNKs population (10) are critical for TLR-mediated response at the decidua is currently unknown.

In malaria endemic populations, single nucleotide polymorphisms (SNPs) within the TLR4 coding and TLR9 promoter regions are associated with variation in disease severity and parasitemia control (87, 88). In the case of pregnancy malaria, primiparous infected mothers with common TLR4 and TLR9 polymorphic variants are correlated with severe complications such as low birth weight and maternal anemia (89). This highlights the importance of studies involving large cohorts of individuals which include genotyping from pregnant mothers living in malaria endemic regions (see section on “Challenges and future perspective”).

### TLRs in Animal Models of Placental Parasite Infection

Animal models have also been used to study the functional role of TLR signaling, particularly for pathogens that are intracellular at some stage of their life cycle (Table 1). TLR4 and TLR9 are strongly activated by malaria parasite PAMPs such as glycosylphosphatidylinositol (GPI), DNA, and hemozoin (90, 91) (Figure 2). In a mouse model of placental malaria, TLR4, and Myd88 signaling activation resulted in placental expression of pro-inflammatory markers, such as IL-6 and TNF-α (92, 93). These studies also demonstrated that malaria parasite infection and inflammation in the mouse placenta lead to reduced fetus growth rate and disorganization of the vascular space in the placenta (92, 93). However, TLR-mediated inflammation and pathology in the human placenta upon malaria infection is unknown and remains to be further investigated.

Studies of congenital toxoplasmosis are also currently limited to animal models. TLR2 and TLR4 are associated with recognition of *T. gondii*'s infection in mice (94). Engagement of the *T. gondii* ligand by TLR2 and TLR4 at the SCT-blood or in the EVT-decidua compartments is plausible, although there is still no direct evidence for such host-parasite interaction in humans. TLR11 has a role in controlling *T. gondii* infection in mice (95, 96), however in humans TLR11 is a pseudogene and is not expressed (97).

## Cytosolic Pattern Recognition Receptors: RIG-I, MDA5, and Nod-Like Receptors at the Uterine-Placental Interface

Cytosolic PRRs play an important role in fighting against viral infection by eliciting host type I interferons (IFN) antiviral response through recognition of single and double stranded RNA (ssRNA and dsRNA) (98, 99). Examples of PRRs are the cytosolic retinoic acid-inducible gene-I-like (RIG-I) and the melanoma differentiation-associated protein 5 (MDA5) receptors, both expressed in the SCT and VCT of term placenta (100). In the human placenta, there is limited information on the function of RIG-1 and MDA5, but they may play a crucial role in recognizing a variety of RNA viruses, including ZIKV and dengue virus (101).

The Nucleotide binding Oligomerization Domain-like receptors (NOD-like receptors; NLR) recognizes intracellular pathogen products which have entered into the host cytoplasmic compartment (74). Both NOD-1 and NOD-2 receptors, which are known to detect intracellular bacterial peptidoglycans (102), are expressed in the SCT in the first trimester and term placentas (103, 104). The NLR pyrin-containing 1 and 3 proteins (NLRP1 and NLRP3) form the major inflammasome complexes, which contribute to activation of inflammatory caspases and pathogen clearance (105, 106).

Activation of NLRP3 and AIM2 inflammasomes, together with high expression of IL-1R, IL-1β, and caspase-1 was recently shown in the placental tissue of mothers infected with *P. falciparum* with significant pathology (107). In a murine model of intra-amniotic inflammation induced by bacterial LPS, tissue sections from the decidua basalis region expressed high levels of NLRP3, but negligible caspase-1 activation suggesting a possible non-canonical activation of the NLRP3 inflammasome (108). Our analysis shows that decidual dM1 expresses high levels of *NLRP3* transcript at steady state compared to other cell types (10) (Figure 4), thus dM1 may play a role in NLRP3-mediated pathogen recognition during early pregnancy.

## Secreted Host Defenses at the Uterine-Placental Interface

### Antimicrobial Peptides

AMP secreted by epithelial and immune cells are small peptides that bind and destroy most groups of pathogens—bacteria, yeasts, fungi, and viruses (109). In addition to direct killing of pathogens, AMPs can rapidly modulate innate host immune responses by recruiting myeloid cells and lymphocytes to the site of infection and mediating activation of TLR (110, 111). The human placenta expresses high levels of β-defensins, a family of broad spectrum antimicrobial peptides which participate in direct bactericidal and anti-viral activity (112). Specific subtypes of β-defensins (HBD-1, 2, and 3) are expressed in SCTs (112), suggesting these AMPs can target potentially bacterial or viral infection from the maternal blood.

### Antiviral Interferons

Recognition of PAMPs by PRRs during infection leads to production of pro-inflammatory cytokines that can aid in clearing the pathogen (74). Studies on the direct role of pro-inflammatory cytokines on the placenta in the case of infection is limited. Inflammatory mediators can directly influence infection outcome and fetal development, but they can also cause damage to the placenta if produced in excess (113). Amongst the proinflammatory cytokines associated with uterine-placental infection during pregnancy, the antiviral IFN are the most well-characterized.

IFNs are secreted by a variety of cell types as the first line of defense against viral infection (114). Type I IFNs, including IFN-α and IFN-β, are potent antiviral cytokines. IFN-α and IFN-β bind to the IFNAR1/2 receptor and lead to expression of IFN stimulated genes (ISGs), which control virus infection through a variety of mechanisms (114). Loss of IFNAR in the placenta leads to vertical transmission and fetal mortality in murine herpesvirus-68 (MHV68) infected mice (115). In the mouse model of ZIKV infection, type I IFN-mediated signaling is essential for the control of viral replication in the placenta, but can also lead to significant placental pathology and fetal mortality (116, 117). The mechanism of type I IFN-mediated placental pathology has been recently elucidated. IFN-induced transmembrane (IFITM) protein, which normally blocks viral entry into host cells, impairs syncytin-mediated fusion of VCT to form SCT, leading to aberrant placental development (118).

Type II IFN, IFNγ, predominantly produced by NK and CD4+ T cells is crucial in controlling parasitic infection, such as *T. gondii* in mice (94, 119). However, elevated levels of IFNγ in response to *T. gondii* infection can lead to pathological effects during pregnancy including fetal demise (119, 120). Severe placental pathology and fetal death have also been associated with elevation of IFNγ during pregnancy in a murine model of malaria (121). Hence, proper regulation of type I and II IFN-mediated signaling at the uterine-placental interface is crucial in limiting pathogen replication, whilst preserving a balanced environment for normal placental development (122). Type III IFN, IFNλ, are constitutively secreted by the human SCT, which presumably confers antiviral effects against ZIKV infection (123–125).

## Intracellular Host Defenses at the Uterine-Placental Interface

### Tryptophan Metabolism by IDO

Indoleamine 2,3-dioxygenase (IDO) is a host intracellular enzyme which metabolizes the amino acid tryptophan (126). IDO has been associated with maternal immunoregulation during pregnancy (127). It also plays a key role in the control of bacterial and viral replication, through limiting the bioavailability of tryptophan (128). IDO also inhibits the replication of several parasitic pathogens including *T. gondii* in human fibroblasts (129) and *Leishmania spp* in human macrophages (130). Mouse infection with *L. monocytogenes* showed that IDO is elevated in an IFN-γ-dependent manner in stromal cells of the metrial gland and decidua basalis; a crucial process to resolve bacterial infection in the mouse placenta (131). Our findings also show *IDO1* expression is enriched in epithelial glandular and DC1 cell type in the first trimester decidua (10) (Figure 4). The presence of IDO in decidua suggests that the enzyme might have a central role in limiting parasitic, viral, and bacterial replication, thus preventing their spread to the fetus.

## Challenges and Future Perspectives

Research on how the human placenta safeguards itself against infections is challenging due to obvious logistical and ethical issues in obtaining tissue from early in gestation (Box 1). Although animal experimental models have provided important insights relating to the immune responses to pathogenic infection, major differences between human and animal placentas must be considered (30, 31). Likewise, differences between strains of pathogens adapted for mice compared with human clinical isolates should be taken into account as this may lead to variation in pathogenesis and cellular response. One such example is the use of mouse CMV, which is unable to cross the mouse placental barrier, unlike the HMVC counterpart which can be transmitted transplacentally in humans (132). Therefore, all data obtained from studies of infection in pregnant animals needs careful interpretation and consideration prior to translation to clinical infection in humans.

Box 1 **Perspective of vertical transmission and innate immune function during pregnancy and infection.**

A variety of maternal infections can lead to vertical transmission (Table 1). The exact mechanisms these pathogens use to escape host defense and cross the placental barrier into the fetal compartment are not entirely known. Experimental models that recapitulate infection of the human placenta and thus vertical transmission are challenging to set up. More data and representative experimental models are needed to answer these questions: (i) how do different pathogens escape or modulate the maternal-fetal host innate immune barrier (ii) why do some pathogens lead to congenital infection but not others? Studying infected human placentas will be essential in understanding this but access to these samples is difficult especially in low and middle-income countries (LMIC) where maternal infection is particularly prevalent (WHO, Maternal mortality index 2019). Despite evidence of expression in primary placental tissue, functional studies on important innate immune features such as TLRs, AMPs, RIG-I, MDA5, NLRs, and IDO during infection and pregnancy are lacking. Understanding how different cell types at the uterine-placental interface (HB cells, dNKs, and dMs) respond to pathogen challenge is essential, but remains under-researched. A critical obstacle is to also extrapolate the protective and pathological mechanisms of cytokines from mouse to human infection. Therefore, systematic comparison of the innate immune effector mechanisms across gestation, in the placenta and decidua from natural human infection vs. healthy pregnancy, will provide a more accurate representation in clinical settings.

Inherent properties of trophoblast cell lines, primary cultures or explants vary between donors, and are likely to be confounded by the area of the placenta that is sampled and as well as stage of gestation (133). For instance, villous placental explants will vary depending on the types of villi sampled and the presence of attached decidual tissue (133). Caution is therefore needed when interpreting data using these experimental models.

To overcome such limitations, population-based cohort studies of women with infection during pregnancy with extensive tissue sampling should be performed. These need to include and focus on LMIC where infection is still a major cause of maternal and fetal mortality and morbidity. Cohort studies and epidemiological surveillance on maternal infections can offer significant insights into disease pathogenesis and accelerate clinical interventions (134). Collaborations between clinicians and researchers for population-based cohort collection and sample processing will be instrumental to achieving this goal. Biological samples such as blood or placenta collected from controls and infected pregnant individuals could be stored and cryopreserved retrospectively. To capture the overall heterogeneity of infected and non-infected placenta samples, sampling, and biobanking criteria of different regions of placenta should be considered (135). Protocols are now available to use frozen tissue processed for single-cell/nuclei and spatial genomics (136, 137). Hence, application of single-cell “omics” on infected vs. healthy human placental and decidual samples will enable us to evaluate cellular heterogeneity in response to infection.

The capacity to detect transcripts specific to host or pathogen mRNA from the same tissue using *in situ* nucleic acid hybridization methods will provide direct quantification of infection burden and identification of potential target host cells within the same tissue (138). Recent advances in spatial transcriptomics methods have also allowed gene expression signatures to be quantified and resolved from individual tissue sections (139). Combination of these emerging technologies with new methods to integrate single-cell and spatial data computationally (140) will provide an unbiased approach to characterize and profile the transcriptome of individual cells *in situ* from the placenta and decidua in response to infections. We anticipate that high-throughput datasets generated from cohort sampling studies will unravel novel cell states and tissue spatial localization associated with placental infections and inflammation. This will also allow us to better characterize not only the innate immune response or makers of infection, but also other adaptive immune states in the human placenta (Box 1).

The use of *in vitro* models will also further define host responses to infection. The recent generation of human trophoblast stem cells (hTSCs) (141) and three-dimensional (3D) trophoblast organoids (142, 143) offer a great opportunity to study infections in early pregnancy where the access to first trimester placental samples is a concern. More importantly, the hTSCs and trophoblast organoids fulfill the criteria characteristic of human first trimester trophoblast *in vivo* (32). Both hTSCs and trophoblast organoids can differentiate *in vitro* into SCT and EVT with appropriate media (142, 143) allowing infection experiments on both the major trophoblast subpopulations present at the two major sites of contact between maternal and fetal cells. Sequencing of both host and pathogen transcriptomes from infected trophoblast at single-cell resolution will also advance our understanding on host-pathogen interactions in placentas (144, 145).

Further refinement of the trophoblast organoid and hTSCs culture system is needed to address key biological questions unanswered by current models. These include studying the effect of infection on cellular crosstalk between trophoblast and other primary placental cells such as HB cells, or decidual cells in culture, such as dNK or decidual stromal cells. Adaptation of CRISPR/Cas9 genome editing technology for the trophoblast organoids or hTSCs will offer novel insights into essential host genes required for vertical transmission and placental defense mechanisms in humans.

## Conclusion

Major maternal and fetal complications as a result of infection are still a concern, especially in LMIC with highest prevalence reported in countries of sub-Saharan Africa (WHO, Maternal mortality index 2019). Profound limitations on current study models and ethical regulations on studying human placenta have significantly delayed the development of therapies and vaccines for maternal-fetal infection. How vertical transmission occurs and how the uterine-placental innate immune system reacts to infection remain as major unresolved questions. Revolutionary advances in single-cell genomics, imaging, computational, and stem cell biology methods are currently underway to study the molecular and cellular mechanisms of human diseases. Therefore, it is now an exciting time to apply these transformative technologies to comprehensively address fundamental questions on host-pathogen interaction at the human uterine-placental interface.

## Author Contributions

RH, AN, and RV-T wrote and edited the manuscript. All authors contributed to the article and approved the submitted version.

## Conflict of Interest

The authors declare that the research was conducted in the absence of any commercial or financial relationships that could be construed as a potential conflict of interest.
